# National Assessment of Data Quality and Associated Systems-Level Factors in Malawi

**DOI:** 10.9745/GHSP-D-17-00177

**Published:** 2017-09-27

**Authors:** Richael O'Hagan, Melissa A Marx, Karen E Finnegan, Patrick Naphini, Kumbukani Ng'ambi, Kingsley Laija, Emily Wilson, Lois Park, Sautso Wachepa, Joseph Smith, Lewis Gombwa, Amos Misomali, Tiope Mleme, Simeon Yosefe

**Affiliations:** a Department of International Health, Johns Hopkins Bloomberg School of Public Health, Baltimore, MD, USA.; b Central Monitoring and Evaluation Division, Ministry of Health, Lilongwe, Malawi.; c Ministry of Lands, Housing and Urban Development, Lilongwe, Malawi.; d National Statistical Office, Zomba, Malawi.

## Abstract

**Background:**

Routine health data can guide health systems improvements, but poor quality of these data hinders use. To address concerns about data quality in Malawi, the Ministry of Health and National Statistical Office conducted a data quality assessment (DQA) in July 2016 to identify systems-level factors that could be improved.

**Methods:**

We used 2-stage stratified random sampling methods to select health centers and hospitals under Ministry of Health auspices, included those managed by faith-based entities, for this DQA. Dispensaries, village clinics, police and military facilities, tertiary-level hospitals, and private facilities were excluded. We reviewed client registers and monthly reports to verify availability, completeness, and accuracy of data in 4 service areas: antenatal care (ANC), family planning, HIV testing and counseling, and acute respiratory infection (ARI). We also conducted interviews with facility and district personnel to assess health management information system (HMIS) functioning and systems-level factors that may be associated with data quality. We compared systems and quality factors by facility characteristics using 2-sample *t* tests with Welch's approximation, and calculated verification ratios comparing total entries in registers to totals from summarized reports.

**Results:**

We selected 16 hospitals (of 113 total in Malawi), 90 health centers (of 466), and 16 district health offices (of 28) in 16 of Malawi's 28 districts. Nearly all registers were available and complete in health centers and district hospitals, but data quality varied across service areas; median verification ratios comparing register and report totals at health centers ranged from 0.78 (interquartile range [IQR]: 0.25, 1.07) for ARI and 0.99 (IQR: 0.82, 1.36) for family planning to 1.00 (IQR: 0.96, 1.00) for HIV testing and counseling and 1.00 (IQR: 0.80, 1.23) for ANC. More than half (60%) of facilities reported receiving a documented supervisory visit for HMIS in the prior 6 months. A recent supervision visit was associated with better availability of data (*P*=.05), but regular district- or central-level supervision was not. Use of data by the facility to track performance toward targets was associated with both improved availability (*P*=.04) and completeness of data (*P*=.02). Half of facilities had a full-time statistical clerk, but their presence did not improve the availability or completeness of data (*P*=.39 and *P*=.69, respectively).

**Conclusion:**

Findings indicate both strengths and weaknesses in Malawi's HMIS performance, with key weaknesses including infrequent data quality checks and unreliable supervision. Efforts to strengthen HMIS in low- and middle-income countries should be informed by similar assessments.

## Abstract (precis)

Nearly all facility registers were available and complete. But accuracy varied, with antenatal care and HIV testing and counseling performing the best and family planning and acute respiratory infections data less well. Most facilities visibly displayed routine health data and most hospitals and district health offices had staff trained in health management information systems, but training was lacking at the facility level as were routine data quality checks and regular supervision.

## INTRODUCTION

National health management information systems (HMISs) collect data on routine health activities in a country's health system, and are one of the 6 building blocks of a health system. The World Health Organization (WHO) defines a well-functioning HMIS as one that “ensures the production, analysis, dissemination, and use of reliable and timely information on health determinants, health system performance, and health status.”^1^

High-quality data on the services provided by health facilities are necessary to make informed decisions regarding resource allocation, planning, and programming. However, this potentially rich source of data is often overlooked in low- and middle-income countries (LMICs), because it is assumed to be of limited completeness, timeliness, representativeness, and accuracy.^2^ Low confidence in the quality of routine health data negatively impacts its use by program managers and other decision makers.^3^^–^^4^

Routinely collected health services data are often overlooked in low- and middle-income countries because they are assumed to be of limited completeness, timeliness, representativeness, and accuracy.

In place of routine health data, governments and development partners tend to rely on data from intermittent surveys that are typically organized and funded by international organizations.^5^ Parallel monitoring and evaluation systems for specific service areas or health conditions may also be established and used by partners to supplement data collected through the HMIS. There are many advantages to using routine data instead of survey data, including power to describe all administrative levels in the country, near-real time accessibility, and reduced cost.^5^^–^^6^

Recognizing the lack of trust in routine health data and the demand for reliable health data by donors and governments, in 2010 the heads of 8 multilateral and private organizations called on governments and partners to invest in improved information systems for health.^7^ At the Summit on the Measurement and Accountability for Results in Health, held in 2015, the World Bank, U.S. Agency for International Development (USAID), WHO, and other partner organizations issued a “Five-Point Call to Action” that, among other things, called for adequate investment in health information and statistical systems in all countries by 2030.^8^ The data collection mechanisms used by HMISs in LMICs may be of varying quality due to human error, measurement error, or missing values. The WHO recommends that data quality assessments (DQAs) be carried out regularly to assess HMIS performance. Findings from DQAs can be used to understand the strengths and weaknesses of routine health data and the HMIS, and can help to determine the reliability of this information.^9^

WHO and other partners have called for adequate investment in health information systems in all countries by 2030.

Findings from previous studies of HMIS functioning in LMICs provide insight into the current status of data quality in developing countries. A review of DQAs conducted in 23 countries using the Performance of Routine Information System Management (PRISM) framework developed by MEASURE Evaluation found that a lack of standardization of data management procedures contributed to poor data quality in many countries.^10^ Data quality—including the accuracy, timeliness, and completeness of data—ranged from 34% to 72% and use of data for decision making ranged from 30% to 53%.^10^ In one review of immunization data from 41 low-income countries, summary reports included less than 80% of immunization data recorded in patient registers.^11^ A systems assessment of the HMIS in Benin found several organizational factors that were associated with better data quality, including availability of material resources for HMIS activities and supervision for HMIS within the past 6 months.^12^ Two previous DQAs conducted in Malawi found poor data accuracy between facility and community registers and reports, and identified weaknesses in quality controls, among other systems factors in need of strengthening.^13^^–^^14^

Malawi is a low-income country located in sub-Saharan Africa with a population of approximately 17 million.^15^ After suffering from a human resources emergency from the mid-1990s to 2009,^16^^–^^17^ Malawi's health system has now begun to rebound. Life expectancy at birth increased from 43 years in 1994 to 63 years in 2014.^15^ In addition, HIV incidence has dropped: in the early 2000s, Malawi had one of the highest rates in the world, with an estimated 14.2% of adults aged 15–49 infected, whereas today the estimated adult prevalence stands at 9.1%.^18^ Malawi has also demonstrated great progress in reducing the number of new HIV infections among children: between 2009 and 2015, estimated HIV infections in children dropped by 71%, and 80% of pregnant women with HIV now access antiretroviral treatment to prevent mother-to-child transmission.^19^ The infant and under-5 mortality rates have also dropped precipitously since 1990, and Malawi now has lower mortality rates among infants and children than many of its neighbors in the Africa region. Infant deaths within the first year of life fell from 143 per 1,000 live births in 1990 to 43 per 1,000 in 2015, and under-5 deaths dropped from 245 per 1,000 live births in 1990 to 64 per 1,000 in 2015.^20^ Communicable diseases continue to be the leading cause of death,^20^ and with only 46% of women receiving the recommended 4 antenatal care visits,^21^ significant progress is still needed to improve the health status of Malawi's population.

All HMIS and monitoring and evaluation (M&E) activities for the Ministry of Health of Malawi are overseen by the Central Monitoring and Evaluation Division. Each district health office has an HMIS officer, seconded from the National Statistical Office, to oversee the collection and reporting of data from the district to the central level. In 2010, Malawi adopted the District Health Information System 2 (DHIS 2) software for its HMIS. DHIS 2 is a web-based, open-source information system used at the district and central levels.^22^ Most facilities continue to use paper forms to collect and report data to the district level; however, electronic registers have been gradually introduced in limited facilities, beginning in 2008. Malawi recently piloted a data quality application developed by WHO and Statistics Norway within DHIS 2, which allows trained users of the system to monitor completeness of data and to depict time trends that may indicate under- or overreporting of data and/or a real increase in reported cases.

Recognizing a gap in information about the quality of Malawi's routine health data, the government and development partners expressed a desire to better understand HMIS performance. Both the *Malawi Health Sector Strategic Plan 2011–2016* and Malawi's HMIS policy call for regular DQAs.^23^^–^^24^ The DQA described in this article is the first nationally representative DQA to have been carried out under these plans at the health center, hospital, and district levels. This assessment aimed to characterize the quality of routine data generated by Malawi's health sector and to elucidate systems-level factors that may be associated with data quality, with the goal of informing improvements in data quality and increasing use of routine health data.

This article reports on the first nationally representative data quality assessment we are aware of conducted at multiple service levels in Malawi.

## METHODS

### Study Setting and Design

We conducted this study in July 2016 using stratified random 2-stage sampling. We first selected, at random, 3 districts from each of Malawi's 5 zones. We then randomly selected 25% of health centers, excluding village clinics and dispensaries, in each selected district, with a minimum of 4 health centers; this did not include the district hospital, which was purposively selected. For one of the districts that was selected, the Ministry of Health divides it into 2 separate administrative units; we therefore included both administrative units, bringing the total districts selected to 16. In 3 of the selected districts, there was no district hospital; therefore, the rural hospital was purposively selected instead. Data were also collected in the district health office (DHO) of every selected district.

Dispensaries and village clinics were excluded because they provide fewer services than health centers, and the data from these facilities are often aggregated and reported to the nearest health center prior to submission to the district. We also excluded facilities that serve primarily police and military, because access to these facilities is restricted and because these facilities do not provide all services included in this assessment, such as diagnosis of acute respiratory infections. In addition, the 4 central (tertiary-level) hospitals located in Blantyre, Lilongwe, Mzuzu, and Zomba, and the national mental hospital, also located in Zomba, were excluded because they have their own data structures and reporting tools that are not generalizable to other facility types, and because they do not provide all of the services that were included in this DQA. We included all facilities under Ministry of Health auspices, including those managed by the Christian Health Association of Malawi (CHAM) and Adventist Health Services because they are supposed to collect and report data in the same manner as government-managed facilities. While many private facilities do report their routine health data to the Ministry of Health, it was not possible to collect data at these facilities because they are not directly governed by the Ministry of Health and access is thus restricted. Therefore, they were excluded from the sample.

### Data Collection

We adapted a set of Data Quality Review tools developed by WHO, in conjunction with the Global Fund to Fight AIDS, Tuberculosis and Malaria; Gavi; and USAID/MEASURE Evaluation.^9^ We added 31 questions on display of information, data use, and supervision from MEASURE Evaluation's PRISM tools.^25^

Our data collection instrument was comprised of 2 sections. The first section included 15 data verification questions for antenatal care, family planning, and HIV testing and counseling, and 17 data verification questions for acute respiratory infection. At facilities, recounts from the registers for 4 service areas of interest were compared with the total included on the facility's monthly report for 3 months (March, April, and May 2016). The 4 indicators of interest were:

1. Number of pregnant women who completed 4 ANC visits (ANC)
2. Number of units of injectable contraceptives (Depo-Provera) administered (FP)
3. Number of positive HIV tests 1 and 2 (defined as 2 consecutive, positive tests) (HTC)
4. Number of acute respiratory infection (non-pneumonia) cases in children under-5 (ARI)

These indicators will henceforth be referred to by their abbreviated name or acronym. At DHOs, we compared monthly report totals for all facilities in the district with the total in DHIS 2 for the same indicators during the same time period.

We assessed the quality of data in 4 service areas: antenatal care, family planning, HIV testing and counseling, and the outpatient department (with a focus on acute respiratory infection data).

The second section of the assessment tool was a systems assessment. This assessment consisted of an interview with the facility in-charge or most senior health worker available at health centers and hospitals, and the district HMIS officer at DHOs. This interview was comprised of 113 questions at facilities and 58 questions at DHOs, and explored systems-level factors that may be associated with data quality in 9 areas:

1. Monitoring and evaluation structure and function
2. Indicator definitions and reporting guidelines
3. Data collection tools and reporting forms
4. Display of information
5. Internal data quality checks
6. Supervision
7. Data maintenance and confidentiality
8. Data use
9. Use of computerized registers

### Data Analysis

We analyzed data quality across 3 dimensions: availability, accuracy, and completeness.^26^

Data quality was analyzed across 3 dimensions: availability, accuracy, and completeness.

The **availability** dimension assessed the availability, at time of assessment, of registers and reporting forms for ANC, FP, HTC, and ARI for March, April, and May 2016. Each service area uses a separate register and a separate reporting form. FP, HTC, and ARI data are required to be reported to the DHO within 5 days after the last day of the month. ANC visits are reported to the DHO 6 months after the first ANC visit. This interval was based upon national survey data demonstrating that most women in Malawi do not begin ANC until the second trimester of their pregnancy.^19^ Therefore, the 6-month delay increases the likelihood that reports will capture the total number of ANC visits that a woman completes during her pregnancy. For this DQA, the September, October, and November 2015 ANC registers were reviewed and compared with the March, April, and May 2016 reports for this service area.

The **completeness** dimension evaluates the extent to which registers and reports include all data of interest. A register or report was considered complete if it had data recorded for the entire month, without any known days of service provision completely without data.

Some facilities did not provide all services of interest; therefore, both availability and completeness are represented as a percentage of all documents that are expected. Denominator values ranged from 18 to 24 (no selected facility provided fewer than 3 of 4 service areas of interest).

**Accuracy** at facilities was calculated as a “verification ratio,” defined as the ratio between the data collector's recount of the 3-month register total and the sum of the monthly report totals for 3 months as recorded on the facility copy. At DHOs, verification ratios were calculated using only May 2016 data, in accordance with WHO recommendations^8^; specifically, totals from all monthly reports for May 2016 for the 4 indicators of interest were compared with totals in DHIS 2 for May 2016. In addition, verification ratios were calculated comparing register totals with totals recorded in DHIS 2 to understand the accuracy of data as they are transmitted from the facility, to the district health office, to the central level.

Two-sample *t* tests were performed to test the statistical significance of differences in the availability, completeness, and accuracy of data between facilities, by facility characteristic. Welch's approximation was applied when unequal variance between groups existed.

Systems assessment questions were grouped into functional areas to assess facility HMIS performance. We calculated the percentage of facilities that answered yes to each question. A matrix was then created, in which responses were color-coded to identify “hot spots” for recommended Ministry of Health action. Cut-offs among the levels of the matrix were designed to detect meaningful differences in facility performance (Table 1) enabling the Ministry of Health to prioritize its efforts to improve the HMIS.

**TABLE 1.** Key for Hot Spot Table Coloring Scheme Indicating Systems Assessment Results and Recommended MOH Actions

Percent of Facilities Responding Positively to Question^a^	Corresponding Color	Interpretation
80–100	Green	No specific action recommended. MOH can seek to identify actions that may improve or sustain facility compliance.
60–80	Yellow	MOH should undertake actions to improve compliance. The timing and nature of the action depend on the functional area and how critical the component is to HMIS functioning.
<60	Red	MOH should seek to immediately identify underlying reason for low compliance and undertake action to increase compliance in the short-term.

Abbreviations: HMIS, health management information system; MOH, Ministry of Health.

^a^ “Positively” is defined as responding in affirmation to the question. For most questions, this included only those facilities that answered “yes”; depending on the context of the question, it may also include facilities that answered “partly.” This is indicated in the Results section of the article. Eight questions about stock-outs of registers and reports were worded in the inverse, so “no” answers were considered to be responding “positively.”

Stata/IC 14.2 was used for this analysis (StataCorp, College Station, TX).

### Ethical Review

Data collection was determined by the Institutional Review Boards of the Johns Hopkins Bloomberg School of Public Health and the Malawi National Health Sciences Research Committee to be exempt from full review. However, we felt that it was important to ensure participants understood what they were being asked to do; therefore, we informed them of the aims of the project, what we were asking them to provide, and that participation was optional. We also answered any questions and requested their consent to proceed with the study.

## RESULTS

We selected 90 health centers of 466 total health centers in Malawi; 13 district hospitals of 23 total; 3 rural hospitals of 21; and 16 district health offices in 16 of Malawi's 28 districts. Of these, 73 health centers were managed by the government of a total 340, 16 were managed by CHAM of 108, and 1 was managed by Adventist Health Services of 18 (Table 2). Of the hospitals included, all 13 district hospitals and 1 rural hospital were managed by the government of 48 total; the other 2 rural hospitals were managed by CHAM of 43 total managed by CHAM (Table 2). (There are also other types of hospitals, besides district and rural hospitals, for a total of 113 hospitals in the country.) There were no significant differences between the selected health centers and health centers nationally for mean outpatient attendance (*P*=.40), urban/rural location (majority rural; *P*=.55), or managing authority (*P*=.11). When compared with all hospitals, the selected district and rural hospitals had statistically significantly higher outpatient attendance (*P*=.01) and were statistically significantly more likely to be managed by the government (*P*<.01). But like the rest of the country, nearly all selected hospitals were in rural areas (*P*=.57).

**TABLE 2.** Characteristics of Health Centers and Hospitals Selected for the Data Quality Assessment Compared With All Health Centers and Hospitals in Malawi, 2016

	Selected Health Centers (n=90)	All Health Centers (N=466)	*P* Value	Selected District Hospitals (n=13)	Selected Rural Hospitals (n=3)	All Hospitals (N=113)	*P* Value^a^
Monthly outpatient department attendance, median (IQR) (March–May 2016)	2240^b^ (1371, 3174)	2264 (1371, 3213)	.40	9595^c^ (7276, 14737)	1202 (779, 7032)	5308 (2686, 9331)	.01
Location			.55				.57
Rural, No. (%)	87 (97)	455 (98)		13 (100)	3 (100)	111 (98)	
Urban, No. (%)	3 (3)	11 (2)		0 (0)	0 (0)	2 (2)	
Managing authority			.11				<.01
Government, No. (%)	73 (81)	340 (73)		13 (100)	1 (33)	48 (42)	
CHAM, No. (%)	16 (18)	108 (23)		0 (0)	2 (67)	43 (38)	
Adventist Health Services, No. (%)	1 (1)	18 (4)		0 (0)	0 (0)	22 (20)	

Abbreviations: CHAM, Christian Health Association of Malawi; IQR, interquartile range.

^a^ Comparing combined district and rural hospitals with all hospitals.

^b^ For 18 selected facilities, there were missing outpatient attendance data for at least 1 month.

^c^ For 2 facilities, there were missing outpatient attendance data for at least 1 month

### Data Verification

Median scores and interquartile ranges (IQRs) were calculated for each data quality dimension to reduce the influence of potential outliers. District hospitals and DHOs had median scores that indicated that nearly all facility registers and reports were available and complete, with a median (IQR) availability score of 0.96 (0.88, 1.00) and 0.94 (0.71, 1.16), respectively, and a median completeness score of 0.92 (0.79, 1.00) and 0.99 (0.98, 1.00) (Table 3). Health centers did nearly as well, with a median availability score of 0.92 (0.79, 1.00) and median completeness score of 0.88 (0.71, 1.00). Rural hospitals had lower performance in these areas, with a median availability score of 0.75 (0.71, 0.79) and a median completeness score of 0.75 (0.67, 0.83), indicating that some documents were unavailable and/or incomplete at the time of the DQA.

**TABLE 3.** Data Quality Dimension Scores for Availability and Completeness, by Facility Type, Malawi, 2016

	Health Centers (n=90)	District Hospitals (n=13)	Rural Hospitals (n=3)	DHOs (n=16)
Availability score, median (IQR)	0.92 (0.79, 1.00)	0.96 (0.88, 1.00)	0.75 (0.71, 0.79)	0.94 (0.71, 1.16)
Completeness score, median (IQR)	0.88 (0.71, 1.00)	0.92 (0.79, 1.00)	0.75 (0.67, 0.83)	0.99 (0.98, 1.00)

Abbreviations: DHO, district health office; IQR, interquartile range.

Nearly all facility registers and reports were available and complete in district hospitals, health centers, and district health offices.

**FIGURE 1:**
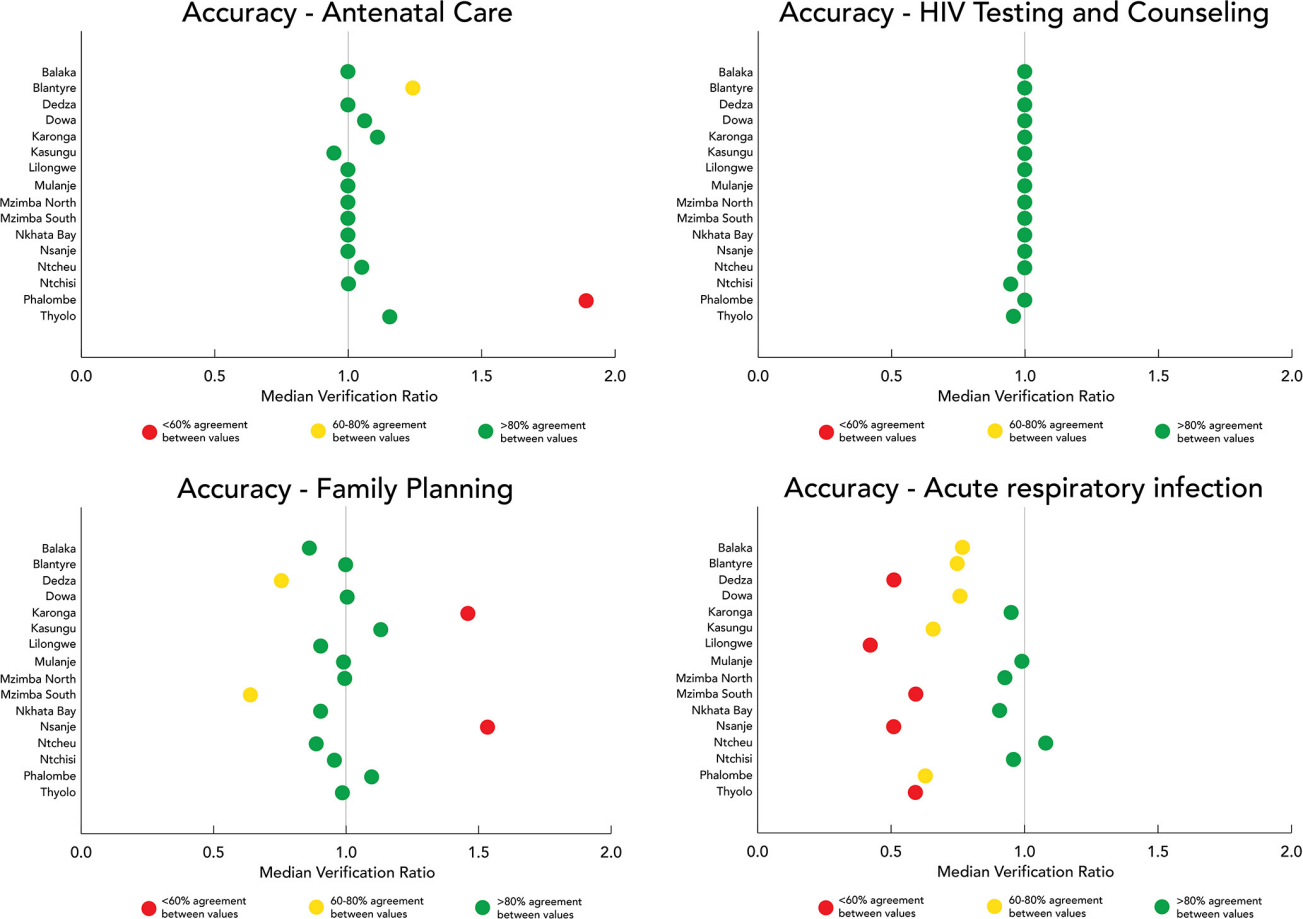
Data Accuracy Verification Ratios for Antenatal Care, HIV Testing and Counseling, Family Planning, and Acute Respiratory Infection Indicators by District, Malawi, 2016

Across the 4 service areas that we assessed, HTC and ANC showed the highest levels of accuracy between registers and reports (Table 4), perhaps because of partner support. In health centers, both ANC and HTC had a median verification ratio of 1.00, indicating that the register and report totals were identical. In district hospitals, the median verification ratio for ANC was also 1.00, although the HTC verification ratio was substantially lower (0.77 [0.61, 0.93]). At rural hospitals, the median score for HTC was 0.99 (0.93, 1.00) and for ANC, 1.08, but ANC verification ratios at rural hospitals varied widely, from 0.00 to 2.50. The family planning indicator showed similarly high accuracy scores, with a median of 0.99 (0.82, 1.36) at health centers and 0.93 (0.80, 1.08) at district hospitals. Only 1 rural hospital that was selected provided family planning services; the verification ratio at that hospital was 0.23. The ARI indicator showed the lowest accuracy scores, ranging from a median of 0.08 (0.07, 0.42) at rural hospitals to 0.87 (0.33, 1.18) at health centers. Substantial variation was seen in district verification ratios for family planning and ARI (Figure 1), but with a few exceptions all districts showed good consistency between registers and reports for ANC and HTC indicators.

**TABLE 4.** Data Accuracy Verification Ratios Comparing DHIS 2 to Facility Registers and DHIS 2 to DHO Reports by Facility Type, Malawi, 2016

	Facility Registers			DHO Reports to DHIS 2 (n=16)	Facility Registers to DHIS 2 (n=106)
	Health Centers (n=90)	District Hospitals (n=13)	Rural Hospitals (n=3)		
ANC ratio, median (IQR)	1.00 (0.97, 1.13)^a^	1.00 (0.88, 1.06)	1.08 (0.00, 2.50)	1.00 (0.98, 1.13)	1.00 (0.96, 1.10)
FP ratio, median (IQR)	0.99 (0.82, 1.36)^b^	0.93 (0.80, 1.08)	0.23^c^	1.00 (0.95, 1.08)	0.94 (0.70, 1.07)
HTC ratio, median (IQR)	1.00 (0.99, 1.05)	0.77 (0.61, 0.93)	0.99 (0.93, 1.00)	1.00 (0.96, 1.01)	1.00 (0.97, 1.05)
ARI ratio, median (IQR)	0.87 (0.33, 1.18)	0.61 (0.20, 0.94)	0.08 (0.07, 0.42)	1.00 (0.83, 1.00)	0.73 (0.27, 1.05)

Abbreviations: ANC, antenatal care; ARI, acute respiratory infection; DHIS 2, District Health Information System 2; DHO, district health office; FP, family planning; HTC, HIV testing and counseling; IQR, interquartile range.

^a^ n=89.

^b^ n=86.

^c^ Only 1 selected rural hospital provided family planning services.

Antenatal care and HIV testing and counseling service areas had the highest levels of accuracy between registers and reports while acute respiratory infection data had the lowest accuracy scores.

Accuracy between facilities, DHOs, and the central level was assessed through verification ratios between facility registers and DHIS 2, and DHO reports and DHIS 2. For the former measurement, averages were calculated by facility; for the latter measurement, averages were calculated by DHO. Accuracy between DHO reports and DHIS 2 was high, with a median verification ratio of 1.00 for all service areas (Table 4). Accuracy between facility registers and DHIS 2 showed greater variability across service areas: while ANC and HTC had median verification ratios of 1.00, family planning and ARI had lower verification ratios (0.94 and 0.73, respectively).

### Systems Assessment

Findings from the systems assessment were divided into 9 functional areas. Table 5 displays a key finding from each functional area, by level of the health system. For the full details on each functional area, see the Supplement. Overall, less than 60% of health centers, hospitals, and DHOs had adequate performance in 8 of the 10 functional areas. In particular, only about 40% of health centers, hospital, and DHOs performed routine internal data quality checks and regular supervisory visits from the district were lacking (52% of health centers and 63% of hospitals reported regular supervision). The 2 functional areas in which facilities and DHOs demonstrated adequate performance were visual displays of routine health data and staff responsibilities. Over 90% of facilities, and 81% of DHOs, displayed information on maternal health, child health, facility utilization, disease surveillance, and/or demographic characteristics, and/or displayed a map of the facility's catchment area. More than three-quarters (81%) of hospitals and 94% of DHOs (but only 58% of health centers) reported having staff trained in HMIS-related functions. In Table 5, the presence of computerized registers for one or more functional areas was not color-coded because this was not considered to be a measure of facility performance.

**TABLE 5.** Key Findings for Systems Assessment Functional Areas, by Health Systems Level, Malawi, 2016

Functional Area	Indicator	Health Centers (n=90) No. (%)	Hospitals (n=16) No. (%)	DHOs (n=16) No. (%)
Staff responsibilities	Staff members have received training for HMIS-related functions	52 (58)	13 (81)	15 (94)
Indicator definitions	Written definitions for all 4 indicators of interest (ANC, FP, HTC, ARI) available in facility or DHO	39 (43)	12 (75)	9 (56)
Reporting guidelines	Reporting guidelines available at facility that describe what should be reported, how reports are to be submitted, to whom, and when	90 (34)	8 (50)	6 (38)
Data use	Regularly use data to calculate indicators	48 (53)	12 (75)	12 (75)
Registers and reporting forms	No stock-outs of any registers or reporting forms during the past 12 months	23 (26)	6 (38)	-
Registers and reporting forms	Sufficient copies of data collection tools available in the DHO to meet the needs of all health facilities in the district	-	-	7 (44)
Display of routine data	One or more information displays present at time of assessment^a^	83 (92)	15 (94)	13 (81)
Internal data quality checks	Consistency checks of collected data routinely conducted	37 (41)	7 (44)	7 (44)
Supervision	Regular supervisory visits from district	47 (52)	10 (63)	4 (25)
Computerized registers	Facility uses computerized registers	9 (10)	15 (94)	-

Abbreviations: ANC, antenatal care; ARI, acute respiratory infection; DHO, district health office; FP, family planning; HMIS, health management information system, HTC, HIV testing and counseling.

^a^ Evaluated the following displays: maternal health, child health, facility utilization, disease surveillance, map of catchment area, summary of demographic data.

Finally, we assessed differences in data quality dimensions for other facility characteristics that we hypothesized may be associated with data quality (Table 6). In our sample, 50% of facilities employed a statistical clerk; employment of a statistical clerk was not significantly associated with any data quality dimension. More than half (60%) of all facilities reported a documented supervisory visit within the last 6 months, which was associated with lower accuracy of ANC register and report data (*P*=.03) but with a higher level of data availability (*P*=.05). Regular supervision from the central level was associated with a higher HTC verification ratio (*P*=.04). Use of data by the facility to track performance toward targets was associated with both improved availability (*P*=.04) and completeness of data (*P*=.02).

**TABLE 6.** Association of Selected Facility Characteristics With Data Quality Dimensions, Mean (*P* Value)

Facility Characteristic	Data Quality Dimension					
	Availability Score Difference	Completeness Score Difference	Accuracy Difference			
			ANC	FP	HTC	ARI
Partner support	1.27 (.12)	0.02 (.57)	0.04 (.78)	0.11 (.54)	−0.07 (.30)	−0.04 (.82)
Statistical clerk employed	0.03 (.39)	0.01 (.69)	−0.08 (.58)	−0.08 (.65)	−0.07 (.25)	−0.36 (.07)
Managing authority^a^	0.08 (.07)	0.08 (.09)	−0.22 (.15)	−0.58 (.26)	−0.03 (.49)	−0.11 (.71)
Facility location^b^	−0.13 (.25)	−0.09 (.42)	−0.14 (.08)	−0.05 (.84)	−0.18 (.18)	−0.12 (.79)
Regular supervision visits from district	−0.02 (.43)	−0.01 (.78)	0.18 (.16)	0.07 (.67)	−0.08 (.18)	0.22 (.21)
Regular supervision visits from central level	0.04 (.22)	0.04 (.31)	−0.02 (.87)	−0.14 (.47)	0.13 (.04)	0.19 (.33)
Supervisory visit within last 6 months	0.07 (.05)	0.03 (.35)	−0.39 (.03)	−0.09 (.60)	0.06 (.37)	0.21 (.29)
Consistency checks of data routinely conducted	−0.08 (.64)	0.05 (.76)	−0.05 (.45)	0.09 (.14)	−0.15 (.43)	0.14 (.46)
Facility uses computerized registers for one or more service areas	0.02 (.66)	−0.00 (.96)	-	-	-	-
Facility uses computerized registers for designated service area (ANC, FP, HTC, outpatient department)	-	-	−0.23 (.23)	−0.58 (.31)	−0.05 (.77)	−0.32 (.15)
Facility has appropriate and adequate space for secure organization and storage of registers and reports	0.02 (.73)	0.02 (.65)	−0.66 (.18)	0.15 (.62)	0.05 (.65)	−0.08 (.78)
Facility uses its data to track performance toward meeting targets	0.06 (.04)	0.08 (.02)	0.07 (.59)	−0.27 (.13)	0.08 (.24)	−0.03 (.88)
Programmatic decisions taken by the facility are based on analyzed data/results	0.04 (.23)	0.02 (.56)	-0.37 (.13)	−0.21 (.38)	−0.01 (.94)	0.08 (.71)

Abbreviations: ANC, antenatal care; ARI, acute respiratory infection; CHAM, Christian Health Association of Malawi; FP, family planning; HTC, HIV testing and counseling.

^a^ Difference between facilities managed by the government and facilities managed by CHAM or Adventist Health Services.

^b^ Difference between urban and rural facilities.

Use of data by the facility to track performance was associated with both improved availability and completeness of data.

## DISCUSSION

This study, which evaluated the quality of the routine health data generated by Malawi's HMIS, identified both strengths and weaknesses of the system. We found that facilities in Malawi were likely to display their routine health data within the facility and that most hospitals and DHOs have trained HMIS staff. Accuracy between ANC registers and reports, and between reports and DHIS 2, was good. However, we found room for improvement in several areas, including:

1. The availability, completeness, and accuracy of data for family planning, HTC, and ARI services
2. Data quality checks at the facility level
3. The comprehensiveness and reliability of HMIS supervision
4. Staff training for HMIS at the facility level

**FIGURE 2:**
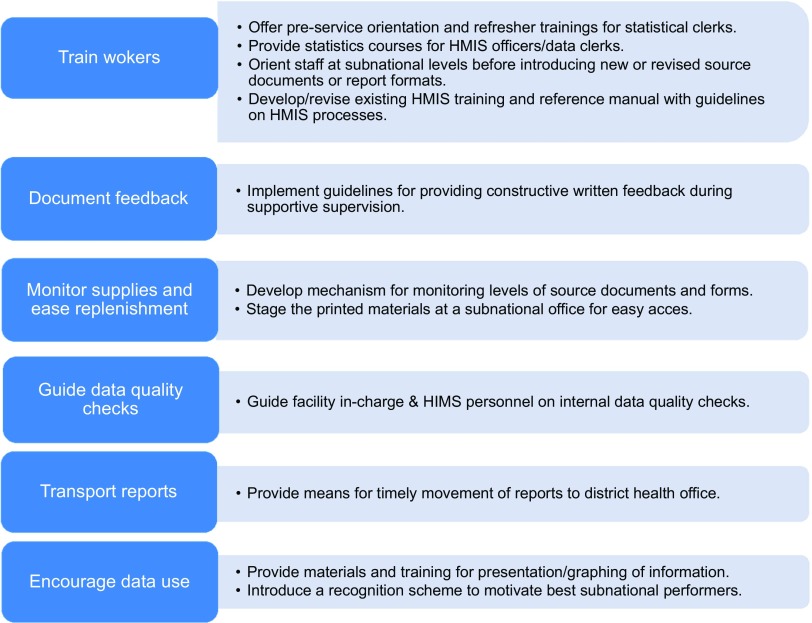
Data Quality Assessment Recommendations to Strengthen the HMIS Abbreviation: HMIS, health management information system. Source: Malawi Ministry of Health (2007).^27^

Identification of these weaknesses provides guidance for HMIS strengthening activities planned by the Ministry of Health of Malawi and development partners (Figure 2).^27^

Facilities and DHOs performed well in selected areas of data quality, particularly in accuracy between registers, reports, and DHIS 2 for the number of women completing 4 ANC visits during the time period of interest. However, data quality was poor in other service areas and dimensions; the availability, completeness, and accuracy of ARI data is in particular need of improvement. Further exploration of the reasons for differences in performance across service areas, including ease of use of the various registers, is needed. Discrepancies between registers and DHIS 2 data for family planning and ARI have implications for program planning and policy at the national level, allocation of resources to the district level, and monitoring of key health indicators. Because these discrepancies were noted between registers and DHIS 2, and not between reports and DHIS 2, we can conclude that issues with facility aggregation, and not district entry of data into DHIS 2, is likely the cause of this discrepancy.

Differences in data quality across service areas should be explored further.

DHOs showed the highest performance in the data quality dimensions. This may be explained by two primary characteristics. Firstly, DHOs are more likely to have trained staff in their offices to carry out data entry and recording activities. In addition, the data entry process is arguably simpler in DHOs than in facilities, because DHIS 2 will automatically calculate monthly summaries, whereas staff in facilities must count and sum individual cases. However, DHOs faced one significant data quality issue. The 75th percentile of availability scores for DHOs was greater than 1 (more than 100%). This is likely because DHOs did not know the number of reports that they should receive from facilities each month, and therefore provided an underestimate of the true figure. It was not possible to independently verify the number of monthly reports that should be submitted each month to the DHO; HMIS officers informed us that the number of facilities providing and reporting services is not constant from month to month, and the Master Facility Register is updated only intermittently, when funding is available from development partners (personal communication with P. Naphini, MOH DHIS-2 Data Manager, National Evaluation Platform-Malawi, June 27, 2017). Therefore, the Ministry of Health should prioritize the identification of the facilities that fall under the jurisdiction of each DHO for the HMIS. This initiative will improve tracking of the availability and completeness of data.

We hypothesized that various functional areas of the health system and facility characteristics were associated with data quality. External partner support, employment of statistical clerks at health centers and hospitals, regular supervision from the district, and the use of computerized registers at the facility were not significantly associated with better performance in any data quality dimension. The Ministry of Health and donors should further examine the lack of association between external partner support and data quality, as it may indicate a need to revisit the effectiveness and appropriateness of partner activities. However, this finding may reflect partners' choices of districts or other confounding factors. The lack of association between employment of a statistical clerk and data quality could suggest that, in addition to other influential factors, statistical clerks may not be adequately trained, supported, or supervised. Therefore, prior to investment in the recruitment of more statistical clerks, as recommended in the National Statistical Strategic Plan, the Ministry of Health should revisit its training and retention strategies for these employees. Finally, while receipt of a supervisory visit within the past 6 months was associated with better data quality, regular district supervision was not. The Ministry of Health should explore strategies for improving supervision, including the use of checklists and joint visits with the zonal or central levels. Facilities may also benefit from regular feedback on their submitted reports, in addition to in-person visits. In the absence of trained facility staff, DHOs should provide analyses of facility data; this will better equip facilities to use their data for planning. DHOs and the central level can also use the WHO data quality app within DHIS 2 to provide an analysis of data quality to the facilities.

Prior to investing in recruiting more statistical clerks, the Ministry of Health should revisit its training and retention strategies for these employees.

**FIGURE 3:**
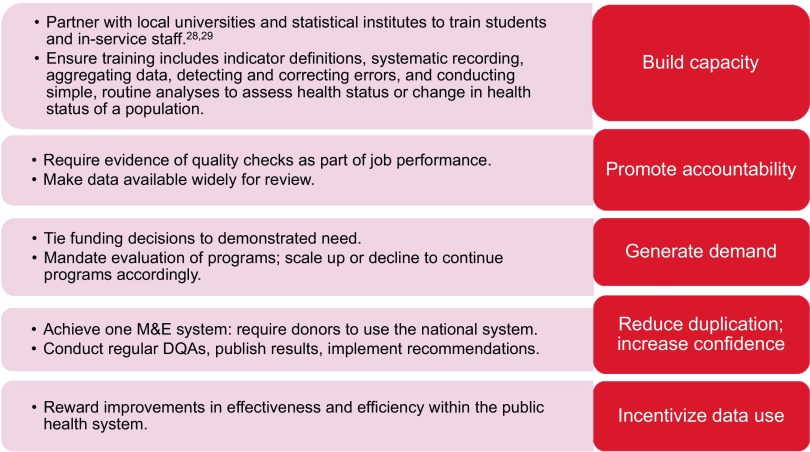
Agenda for Building Country Leadership for Data Use, Malawi

The systems assessment permitted us to evaluate the performance of various HMIS functional areas across facility types. The systems assessment revealed poor performance of health centers, hospitals, and DHOs in most functional areas; display of information was the only exception for all system levels. Disaggregation of facility performance in the various functional areas by district or zone can help the Ministry of Health to prioritize geographic areas for intervention. In addition, our findings of the association of various systems-level factors with data quality may assist the Ministry of Health in identifying the most effective programs for improving quality. Use of data by the facility to track performance was associated with higher availability and completeness of data. This relationship may indicate that facilities that use their routine data were more likely to review those data for these attributes and to place greater value in the quality of these data. Promotion of data use is an important part of the cycle of quality improvement. We have included it in our DQA recommendations^27^ (Figure 2) and have featured it in the agenda for building country leadership for data use (Figure 3).^28^ Use of data was not, however, associated with higher accuracy of data, perhaps because only a small number of facilities reported conducting regular accuracy checks on their collected data. Regular accuracy checks were not statistically significantly associated with improved performance in any data quality dimension. This lack of association may be explained by the quality and content of the internal data checks that facilities perform on a routine basis. Because these routine checks were self-reported, we could not verify their existence or quality; however, at health centers, less than 60% of staff designated to conduct HMIS activities had been trained in HMIS functions. Therefore, they may lack the skills needed to properly assess data quality and to carry out the appropriate checks for accuracy between registers and reports. Training should be implemented to ensure staff members know how to confirm the accuracy and completeness of data.

The findings of the systems assessment allowed us to evaluate compliance with Malawi's HMIS policy, which was introduced in October 2015. Because this DQA was conducted only 8 months after introduction of HMIS policy, these data could serve as a baseline to assess improvement. Repeated DQAs will enable the Ministry of Health to monitor progress of HMIS strengthening activities, as outlined in the HMIS policy. Results of this DQA should be shared with program managers, M&E officers, and other decision makers, in order to provide an empiric measure of data quality in Malawi. Especially if improvements are seen, reporting results of subsequent DQAs could improve the confidence of potential data users in the quality of the routine health data generated by this system.

This DQA complements other DQAs that were previously conducted in Malawi. A DQA conducted for integrated community case management (iCCM) found a generally well-functioning M&E system for iCCM but quality controls were lacking and there were gaps in the completeness and accuracy of reporting by Health Surveillance Assistants.^14^ Our DQA found similar issues at higher levels of the health system and in other service areas. Our findings also build on the findings of a previous study that examined the consistency of Malawi's reporting of under-5 deaths with survey data. This study found that concordance of reporting data with “gold-standard” survey data was low, ranging from 35% to 65% in 2 districts.^13^ In conjunction with the findings of our DQA, these previous studies can be used to paint a clear picture of data quality throughout the health system. From the lowest cadre of health workers to the central level, these 3 studies demonstrate that the accuracy, completeness, and reporting of routine health data is in need of improvement in Malawi.

Nationally representative studies on the quality of DHIS data are rarely published. Findings from this study enrich the existing literature on data quality in low-income countries because they explore data quality across multiple service areas and levels of the health system. In addition, these study findings point to key systems-level factors that influence data quality. With a few exceptions, including DQAs conducted in Liberia and Côte d'Ivoire,^10^ previous DQAs conducted in sub-Saharan Africa have been more limited in their scope, examining only 1 service area, 1 type of health setting, or a limited number of districts. While performance on data quality dimensions are limited in their generalizability to other countries, given the variability of health systems, the findings of this data quality and systems assessment can be used to drive evidence-based improvements in the HMIS of other similar countries. For example, the lack of association between the use of computerized registers at the clinic level and data quality may indicate the need to accompany the introduction of these electronic systems into HMISs with training and data use initiatives. Furthermore, we show here that conducting comprehensive, high-quality data quality and systems assessments is feasible in a low-income country. Other LMICs that wish to gather information about current HMIS functioning to strengthen their HMISs can replicate this assessment methodology.

Findings from this study enrich the existing literature on data quality in low-income countries because they explore data quality across multiple service delivery areas and levels of the health system.

### Limitations

This study had limitations that warrant further discussion, many of which may be addressed in future assessments of this type. Firstly, the median outpatient department attendance at the selected district hospitals was higher than in hospitals of all types in the country, and the median attendance at the selected rural hospitals was lower than in hospitals of all types; therefore, caution should be exercised when generalizing these findings to hospitals of differing sizes. In addition, because the selected hospitals were statistically significantly more likely to be managed by the government rather than the faith-based entity (CHAM), attention should be paid to the management structure of the target hospitals when designing and implementing interventions. We did not power our assessment to allow for comparisons among hospitals. Also, we did not have access to records from village clinics, Health Surveillance Assistants, or private providers during the DQA. This limited our ability to provide a complete picture of data quality, particularly at health centers and district hospitals where data from these providers is included in monthly reports. Thirdly, because we did not include private providers or lower-level health facilities in the DQA, it is important to remember that the findings of this study represent only health centers and hospitals managed by the government, CHAM, and Adventist Health Services, and the district health offices. Finally, this analysis does not address validity and representativeness of routine health data. Validity measures the agreement of routine health data with a “gold standard,” usually defined to be survey data. Representativeness examines how well routine health data reflects the underlying disease state of the population.^5^^,^^9^ These dimensions will be analyzed in later studies through a comparison of these indicators with survey data, after we are able to analyze the 2015 Malawi Demographic and Health Survey data.

## CONCLUSION

Data quality is a multifaceted concept that cannot be boiled down to a binary measure; attempts to improve data quality should consider each dimension of quality. As one of the 6 building blocks of a health system, as defined by WHO, health information interacts with the other areas of the health system, including human resources, financing, and governance. Therefore, interventions to address the quality of data must approach the problem from multiple angles while also considering the systems-level implications of HMIS improvement.

Based on the findings from this study, we recommended that the Malawi Ministry of Health focus on training staff at all levels of the health system in HMIS, improving HMIS-focused supportive supervision, ensuring internal data quality reviews, and encouraging data use to inform programming (Figure 2).^27^. In addition, the National Evaluation Platform's agenda for building country leadership for data use focuses on improving the enabling environment for data improvement and use (Figure 3).^28^^,^^29^

The results of this assessment are already informing decision makers and program managers in Malawi's health sector of ways to improve the use of routine health data in policy and programming. Because the assessment and improvement of data quality is a continuous process, a task force has been named by the M&E Technical Working Group to examine findings and recommendations from this assessment and to develop an appropriate intervention package to address the identified issues. Using these findings, the Ministry of Health and M&E Technical Working Group are working with the Gates Foundation, WHO Health Data Collaborative, and other stakeholders both in-country and internationally to improve data quality and use nationwide.

## Notes

**Peer Reviewed**
